# Correlation between the DNA fragmentation index (DFI) and sperm morphology of infertile patients

**DOI:** 10.1007/s10815-021-02080-w

**Published:** 2021-02-02

**Authors:** Alberto Ferrigno, Giovanni Ruvolo, Giuseppina Capra, Nicola Serra, Liana Bosco

**Affiliations:** 1 Department of Sciences for Department of Biological, Chemistry and Pharmaceutical Sciences and Technologies (STEBICEF), University of Palermo, Viale delle Scienze Ed.16, 90128 Palermo, Italy; 2 Centro di Biologia della Riproduzione, Via Villareale 54, Palermo, Italy; 3 Health Promotion and Mother-Child Care ‘G. D’Alessandro’ (PROSAMI), University of Palermo, Palermo, Italy; 4 Biostatistics Unit - Department of Molecular Medicine and Medical Biotechnology, University Federico II of Naples, via S. Pansini, 80131 Naples, Italy; 5 Department of Biomedicine, Neuroscience and Advanced Diagnostics (Bi.N.D), Section of Biology and Genetics, University of Palermo, 90133 Palermo, Italy

**Keywords:** Human spermatozoa, DFI, TUNEL assay, Pellet Swim up, Sperm morphology

## Abstract

**Purpose:**

To evaluate the correlation between the DNA Fragmentation Index (DFI) and sperm morphology in patients undergoing ICSI, as a predictive parameter in reproductive outcomes.

**Methods:**

A retrospective study was conducted on 125 infertile patients enrolled in a fertility clinic. Seminal characteristics were measured following the WHO guidelines (2010) for the examination of the seminal fluid. After collecting motile sperm population by pellet swim up, DFI was calculated and simultaneously associated with sperm morphology using in situ TUNEL assay and an image analyzer software in at least 250 spermatozoa for each patient.

**Results:**

All subjects were divided into two groups according to a cutoff established, by choice, of the sperm DFI (15%): group A (< 15%) consisting of 65 patients and group B (≥ 15%) of 60 patients. Data were analyzed using non-parametric statistical methods. The results demonstrate that there is no statistical difference between the two groups in seminal characteristics. The collective data show a high significant correlation, suggesting that spermatozoa with abnormal morphology are the best candidates to contain DNA damage (*p* < 0.001). Also, when group A is compared with group B, an increased percentage of morphologically normal spermatozoa with fragmented DNA was observed in patients, with DFI values ≥ 15% (*p* < 0.001).

**Conclusion:**

These results are aimed at providing an exact value of DFI in morphologically normal spermatozoa, which will be helpful to the embryologist in evaluating the risk of transferring, during the ICSI procedure, a spermatozoon whit normal morphology but fragmented DNA.

## Introduction

The infertility condition of a couple is evaluated after 1 year of attempts to obtain a spontaneous pregnancy. Subsequently, one of the first investigations aimed to identify a cause of infertility is the evaluation of the semen sample to define a possible male factor infertility. Literature is consistent in stating that about 50% of infertility cases depend on a male infertility factor [1, 2].

The seminal examination through the evaluation of the traditional seminal parameters, such as concentration, motility, and morphology, despite the strict criteria imposed by the last version of the WHO manual of 2010, can give definitive indications for male infertility when it is derived from azoospermia or globozoospermia [3]. In all other cases, the extreme variability of a seminal evaluation does not allow to assess with certainty the true reproductive capacity of the examined spermatozoa [4]. For this reason, new molecular parameters have been sought to give more information about the quality of the spermatozoon, declined as its ability to fertilize the oocytes and support the embryogenesis to give a full-term pregnancy [5–7].

The human sperm chromatin during spermatogenesis appears to be highly susceptible to structural changes, which also occur as a result of DNA filament cuts, as well as being sensitive to ambient stresses, such as temperature changes, oxidative stress, and environmental pollution, all of them involved in chromatin structure alterations [6]. The ability to repair physiological cuts of sperm chromatin is very efficient during the early stages of spermatogenesis, while it is very limited already in the mature spermatid, due to the strong compaction of the chromatin and the reduction of the cytoplasmic components that prevent its repair [8, 9].

Several studies have shown that a basic requirement for a spermatozoon to be able to successfully fertilize an oocyte and transmit paternal genetic information is the integrity of chromatin contained in the head of the male gamete nucleus [10, 11]. In the oocytes, on the other hand, chromatin integrity is well preserved due to the efficiency of the complex repair machinery of DNA lesions, which is very active in the female gamete [12].

Fertilization, in humans, is a process in which the genetic information contained in the sperm DNA encounters and integrates with maternal DNA within the human oocyte. The integrity of chromatin is an essential requirement in transferring correctly the genetic information to be used for adequate embryonic development capable of producing embryo implantation and a full-term pregnancy [8, 13].

Therefore, chromatin integrity evaluation also appears to be a parameter of the intra-testicular quality of spermatogenesis. Physiologically, during the testicular spermatogenesis, single- and double-strand DNA breaks (DSBs) are made to create cross-overs during meiosis and then, in the round spermatid, to allow DNA compaction by substituting histones with protamines [11, 14]. The DSBs are then revised to restore the integrity of sperm chromatin. However, there is a low physiological amount of spermatozoa that maintains a fragmented chromatin in a seminal sample.

Abnormal and massive fragmentation can occur during the intra-testicular spermatogenesis or in the post-testicular phase, for example, along with the transit in the epididymis, as a result of apoptosis due to an excess of oxygen free radicals, or to bad life habits, such as drug taking, cigarette smoking, and bad working conditions leading to an increase in scrotal temperature, but also as a result of clinical conditions, such as varicocele or exposure to specific environmental pollutants [11, 15–18].

The presence of sperm chromatin fragmentation appears to be associated with a reduction in the reproductive capacity of human spermatozoa and characterizes the semen sample of infertile patients compared to patients with spontaneous fertility [19]. The chromatin fragmentation in spermatozoon appears to be correlated with apoptosis and dysfunction of mitochondrial membrane potential, correlating negatively with some seminal parameters such as sperm motility and morphology [20, 21]. Several studies have shown that the fragmentation in sperm chromatin is partly repaired by the oocyte but negatively interferes with the clinical outcomes after assisted reproductive technologies (ARTs) [22, 23].

The study of sperm DNA fragmentation has had great difficulty getting into the diagnostic routine, because of the variability of the methods used, which have generated confused data also on the interference on clinical results in assisted reproductive techniques. Several methods for analyzing the integrity of sperm chromatin are reported in the literature: from the oldest aniline/toluidine blue staining and protamine examination by chromomycine A3, to the latest techniques, such as TUNEL (terminal deoxynucleotidyl transferase-mediated dUTP nick end labeling), COMET (single-cell gel electrophoresis), SCD (sperm chromatin dispersion), SCSA (sperm chromatin structure assay), DNA ladder, and DNA-break detection FISH (fluorescence in situ hybridization) assays. The advantages and technical limitations of these assays are discussed in different papers [24–26].

Nevertheless, the use of these different methods has not allowed to consider the DFI as a reliable indicator of the reproductive capacity of a semen sample, and its use for diagnostic use is still controversial [27], as well as the choice of sperm population to investigate. In most of the published papers, the DFI evaluation is related to the total sperm population of a seminal sample, while, in our opinion, it would be more useful to investigate it only in the sperm population that will be used for oocyte fertilization in ART cycles, that is, those selected after pellet swim up (PSU)[28].

The PSU is the most used technique in the ART laboratory routine to select the spermatozoa to be used in the oocyte fertilization, and it is based on the ability of the spermatozoa to move through the culture medium, which can be stratified directly on the crude sample or on pellet after centrifugation.

In this retrospective study, we analyzed the DFI using the TUNEL assay on the sperm population recovered after PSU. In addition, we have evaluated the relationship between the DNA fragmentation index and the sperm morphology, because spermatozoa choice for fertilization in ART cycles is done only among the spermatozoa with a normal morphology.

## Materials and methods

This retrospective study was carried out in accordance with the Code of Ethics of the World Medical Association for experiments involving humans [29].

All the patients were evaluated: the DNA fragmentation index (DFI) after PSU and the conventional seminal parameters referred to fresh whole samples. DFI was calculated using the in situ TUNEL assay.

The patients were divided into two groups according to the DFI evaluated in the sperm population isolated after pellet swim up: group A (*n* = 65, mean age 38.2 ± 6.6 years) included those who had a sperm DFI < 15% and group B (*n* = 60, mean age 38.6 ± 6.2 years) included patients with a DFI ≥ 15%. The patients were included in the study only with, at least, 1 million/ml of mobile spermatozoa in the whole sample. Semen samples of all patients were collected after signed informed consent. Patients who met the following exclusion criteria were excluded from the study to avoid interference on the outcomes: diabetes or other systemic diseases, varicocele, prostatitis, fever, medications, recent exposure to X-rays, drug abuse, and job exposures to toxic chemicals. Data were collected by questionnaire.

- Examination of seminal liquid

In accordance with the World Health Organization (WHO) guidelines for the examination of the seminal liquid [30], samples were obtained by masturbation and collected in a clean plastic container (marked with the patient identification number) with a sufficiently large opening.

The samples were maintained in an incubator at 37 °C till the liquefaction process was complete.

For the collection of samples, patients were asked to follow the following rules:

- Sexual abstinence for no less than 48 h and no more than 7 days, in the period preceding the collection procedure;
- Collection of the whole sample to avoid the loss of any ejaculate fraction.

The sample was collected in a specific room near the laboratory.

About 30–60 min after sample production, the appearance, pH, and volume of the semen sample were evaluated.

The number of spermatozoa was estimated using a sperm counting chamber (Makler chamber). Motility was evaluated using a wet preparation approximately 20 μm deep according to the WHO manual and classified as linear progressive sperm motility (PR), non-progressive sperm motility (NP), or immotile (IM). Morphology evaluation was done making semen smears simultaneously with TUNEL assay. After the seminal evaluation, the samples were treated with the pellet swim up, to collect the mobile sperm population on which to perform the DNA fragmentation test.

To carry out the PSU, we used a Kerin modified protocol [28]. Briefly, a semen aliquot was diluted in a 1:2 ratio with the culture medium (Mops, Vitrolife, Sweden) in a tube (FALCON) and centrifugated for 7’ at 300*g*. The supernatants were removed and 1 ml of IVF medium (Vitrolife, Sweden) was gently stratified on the pellets and incubated for 1 h at 37 °C, 6% CO_2_). The supernatant was then aspirated and transferred into an empty tube.

b) Sample preparation for TUNEL assay

**Fig. 1:**
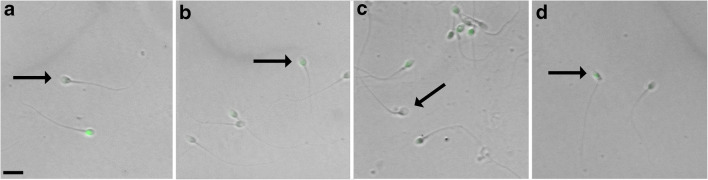
TUNEL assay on spermatozoa samples, images captured by microscopy allows to observe in brightfield the morphology of the sperm, while by green fluorescence, it is possible to visualize, for the same sperm, the presence of DNA fragmentation. Morphologically normal sperm showed non-fragmented DNA (**a**); morphologically normal sperm showed fragmented DNA (**b**); morphologically abnormal sperm (head defect) showed non-fragmented DNA (**c**); morphologically abnormal sperm (neck defect) showed fragmented DNA (**d**). Scale bar = 30 μm

The assessment of sperm DNA fragmentation was performed as described by Ruvolo et al. by TUNEL assay [18]. This method was also used to assess DNA fragmentation in cumulus cells, representing as an indicator of oocyte quality [31–33]. DFI was calculated using in situ TUNEL assay in at least 250 spermatozoa. By means of NIS-Elements BR 3.10 image analyzer software (Nikon) using images of the same field (light, fluorescence, and “merged”), it was possible to evaluate simultaneously sperm morphology associated with DNA fragmentation (Fig. 1).

### Statistics

Data are presented as number and percentage for categorical variables, and continuous data expressed as the mean ± standard deviation (SD) or median with interquartile range (IQR). Test for normal distribution was performed with the Shapiro-Wilk test. In addition, our sample was divided into two subgroups according to an arbitrarily chosen value of DFI; this was used as cutoff (15%); 65 patients (52%) were included in group A (DFI < 15%) while 60 patients (48%) in group B (DFI ≥ 15%). The Mann-Whitney test is used to test the significance of the difference between two independent subgroups. It is the alternative for the independent sample *t* test, when the distribution of the samples is not normal. In order to verify whether there were significant differences among three or more variables, the Kruskal-Wallis test was performed. Particularly if the Kruskal-Wallis test was positive (*p* < 0.05), a post hoc test used for pairwise comparison of variables was performed with the Dunn test and Bonferroni *p* value correction. Finally, the degree of association between two variables was calculated using Spearman’s correlation coefficient rho. All tests with *p* < 0.05 were considered significant. Statistical analysis was performed with the RStudio software for Macintosh (version 1.2.5001).

## Results

The study was conducted between January 2019 and February 2020 (14 months) and included 125 patients undergoing ICSI (mean age 38.3 ± 6.39) and it was realized on motile human spermatozoa, collected after the PSU technique. The sperm DFI was calculated for each patient by examining a sufficient number of fields until 250 sperm cells were counted, a statistically significant number for this type of analysis, in order to evaluate DNA fragmentation related to sperm morphology. The 125 patients were divided into two groups according to the resulting DFI: group A (*n* = 65, mean age 38.2 ± 6.6 years) included those who had a sperm DFI < 15% and group B (*n* = 60, mean age 38.6 ± 6.2 years) included patients with a DFI ≥ 15%. The seminal parameters, performed on fresh whole samples, of patients of group A and group B were respectively: median concentration 20 × 10^6^/ml vs. 18 × 10^6^/ml, percentage of PR motility 20 vs. 30, percentage of NP motility 30 vs. 27.5, percentage of normal morphology 31 vs. 21 (Table 1).

**Table 1:** Semen characteristics of fresh whole samples in patients of group A compared to ones of group B

Sperm parameters	Group A (*n* = 65) Median (IQR)	Group B (*n* = 60) Median (IQR)	Group A vs. group B
Age	38 (34.75, 41.25)	38 (33, 43.75)	0.73 (MW), (rN)
Concentration (× 10^6^/ml)	20 (6, 38.75)	18 (7.5, 35)	0.96 (MW), (rN)
PR motility (%)	20 (20, 34.5)	30 (15, 40)	0.42 (MW), (rN)
NP motility (%)	30 (11.25, 30)	27.5 (17.5, 30)	0.63 (MW), (rN)
Normal morphology (%)	31.2 (9.75,50.25)	21 (12,33)	0.13 (MW), (rN)
DFI (%)	< 15	≥ 15	/

*Significant test (*p* < 0.05); p, *p* value; *SW*, Shapiro-Wilk test for normal distribution; *rN*, reject normality; *IRQ*, interquartile range; *MW*, Mann-Whitney test

No statistical difference was found in any of the seminal parameters evaluated between the two groups. Of the total analysed sperm population, 32% had a normal morphology, while the remaining 68% had at least one morphological alteration. The TUNEL method for the evaluation of sperm chromatin fragmentation allows to observe in brightfield the morphology of the spermatozoon, while by fluorescence, it is possible to visualize, for the same spermatozoon, the presence of DNA fragmentation (Fig. 1). So, with this assay, it was possible to correlate the morphological data to the molecular ones. According to this methodology, we found that 29% of morphologically normal spermatozoa showed non-fragmented DNA (Fig. 1a); 3% of morphologically normal spermatozoa showed fragmented DNA (Fig. 1b); 54% of morphologically abnormal spermatozoa showed non-fragmented DNA (Fig. 1c); 14% of morphologically abnormal spermatozoa showed fragmented DNA (Fig. 1d).

Usually, in an ICSI procedure, a morphologically normal spermatozoon is selected to be injected into the oocyte. Thus, by analyzing only spermatozoa with normal morphology, we found that 91.77% of them were characterized by the absence of DNA fragmentation, whereas the remaining 8.23% were apoptotic spermatozoa.

In order to verify the normality of the three variables of interest, the Shapiro-Wilk test was performed, providing a *p* < 0.005 for all.

Since normality was violated, the non-parametric Mann-Whitney test and Kruskal-Wallis test were used to verify significant differences between the two groups and significant differences among variables into groups, respectively (Table 2).

**Table 2:** Statistical analysis into group A and group B and between the group A and group B

Variables	Group A (*n* = 65) median (IQR)	Group B (*n* = 60) median (IQR)	Group A vs. group B
(1) DFI TOT (%)	9.74 (5.17–11.76)	22.21 (17.73–30.57)	*p* < 0.0001*, (rN)
(2) DFI NORM (%)	1 (0.0–4.0)	8.0 (3.0–14.5)	*p* < 0.0001*, (rN)
(3) DFI ANORM (%)	21 (13.0–29.0)	48 (36.5–62.0)	*p* < 0.0001*, (rN)
Statistical analysis into groups	*p* < 0.0001*, (1) > (2)*, (1) < (3)*, (2) < (3)*, (DB)	*p* < 0.0001*, (1) > (2)*, (1) < (3)*, (2) < (3)*, (DB)	

*Significant test (*p* < 0.05); *p*, *p* value; *rN*, reject Normality; *DB*, Dunn’s test with Bonferroni *p* value corrected post hoc Kruskal-Wallis test; *IRQ*, interquartile range

In line with the aim of the study, a correlation analysis was conducted to understand what kind of relationships between the three different variables there was.

**Fig. 2:**
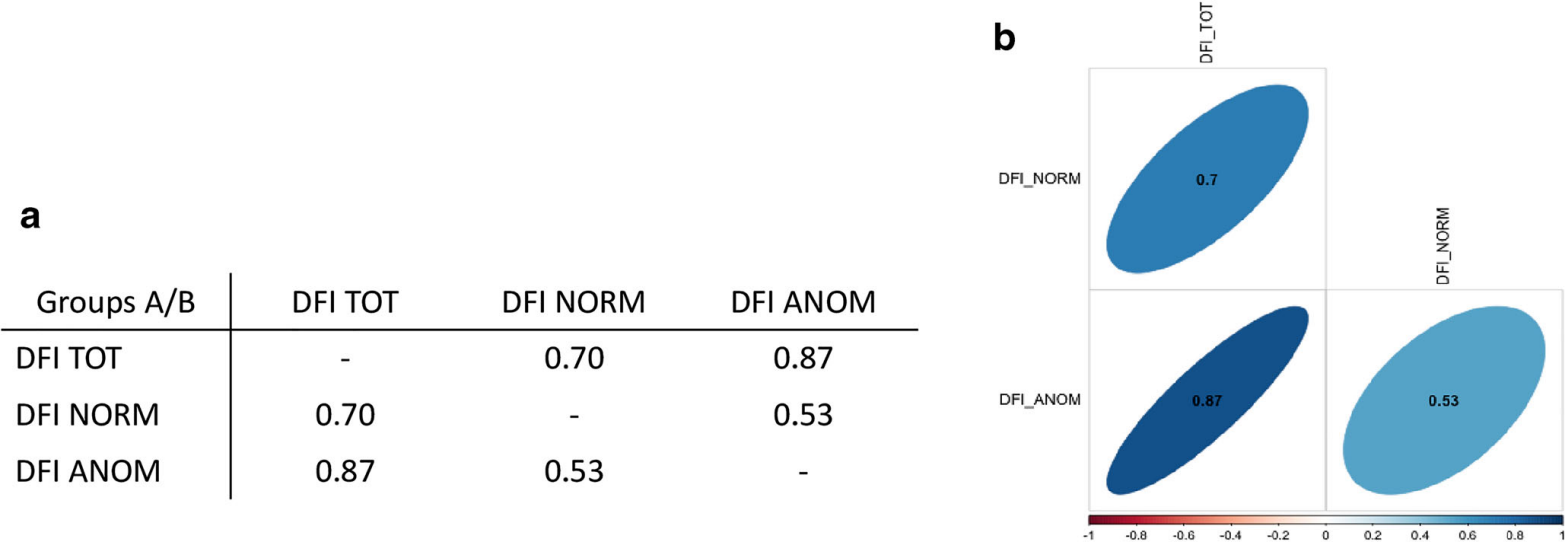
Correlation analysis in two groups showing a positive correlation between the three variables, as indicated by correlation matrix (**a**) and corrplot (**b**)

Thus, a positive correlation was observed among the three variables using Spearman’s rank correlation method, as shown in Fig. 2 by correlation matrix and corrplot.

Therefore, in order to verify the statistical significance of the abovementioned correlations among the two groups (A vs. B), a univariate multiple regression analysis was performed involving the total DFI, as an independent variable, the DFI of morphologically normal spermatozoa and that of morphologically abnormal ones as dependent variables.

**Fig. 3:**
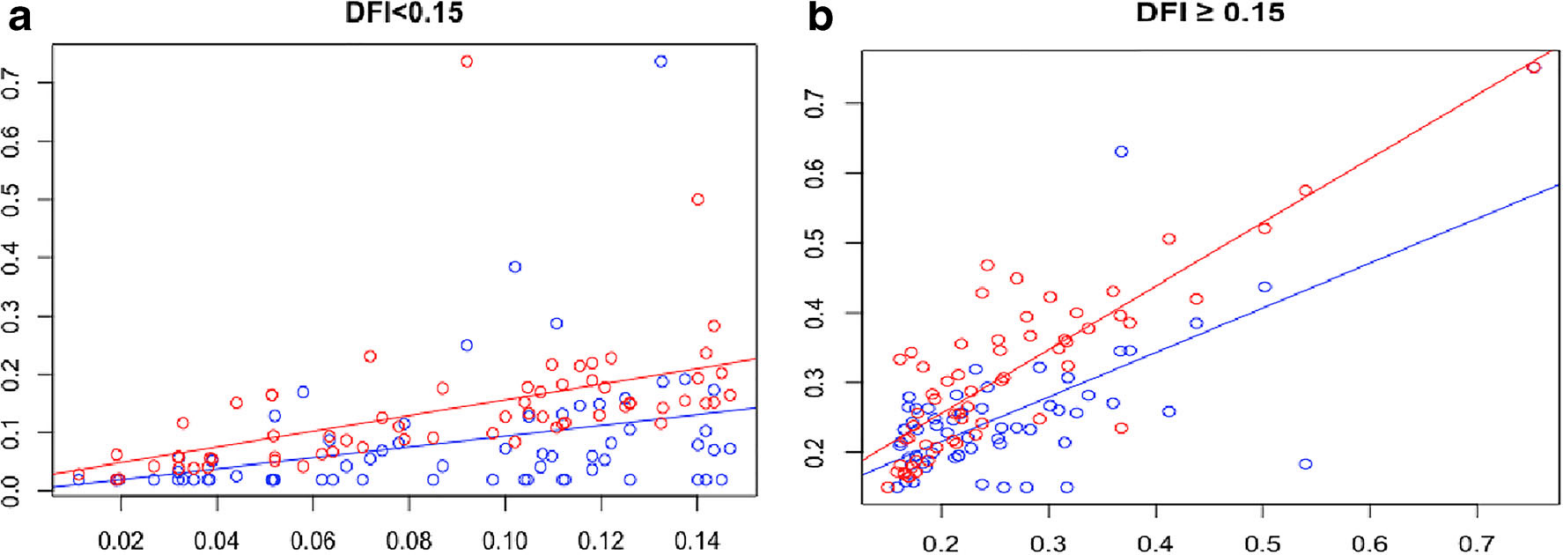
Univariate multiple regression analysis among two groups performed using DFI NORM (blue points) and DFI ANOM (red points), as dependent variables (on the y axis), and DFI TOT as an independent variable (on the x axis). DFI values are reported as decimals. **a** showed the results of group A and in **b** of group B

The results are given in the panel of Fig. 3.

Total DFI was significantly more correlated with the DFI of morphologically abnormal spermatozoa (*r*_s_ = 0.73, *p* < 0.001) than that of morphologically normal ones (*r*_s_= 0.45, *p* = 0.005) in group A (Fig. 3a). This suggests that in a seminal sample, the spermatozoa with abnormal morphology are the best candidates to contain damages into DNA.

Furthermore, in group B, total DFI showed a significant positive correlation with the DFI of morphologically normal spermatozoa (*r*_s_ = 0.44, *p* < 0.001) and the DFI of morphologically abnormal spermatozoa (*r*_s_= 0.80, *p*< 0.001), as reported in Fig. 3b.

Although abnormal spermatozoa showed a higher sperm DFI than spermatozoa with normal morphology, a comparison of slopes of regression lines between the normal sperm DFI (Fig. 3 a and b: blue lines) allowed to affirm that, at the same variation of total DFI, there was a greater increase of normal sperm DFI in group B than in group A.

## Discussion

Our study evaluated the possibility of using the sperm DFI as an indicator of the fertilizing capacity of a seminal sample in assisted reproductive technologies (ART). In particular, we tried to verify if it can be more effective, for this purpose, to evaluate DFI not on the total sperm population of a seminal sample, but on the mobile sperms, with normal morphology, isolated after swim up pellets, among which, in the clinical routine, the embryologists select the sperm to be injected in the ICSI procedure.

A recent meta-analysis by Cissen et al. considers that among all the methodologies used for the evaluation of the sperm DFI, the tests SCSA and SCD are those with low predictive capacity, unlike TUNEL and comet assay, which, being direct techniques, are capable of greater sensitivity and specificity than the former methods [11].

The negative interference of sperm chromatin fragmentation on subsequent embryogenesis and clinical outcome after IVF or ICSI appears controversial. Several authors have shown that the oocyte, with its molecular repair system, tends to adjust the discontinuity of the sperm chromatin already from the early stages of fertilization, during the zygote stage [34, 35]. Indeed, Perez-Cerezales et al., using trout oocytes have shown that the repair activity appears to be limited in cases where sperm damage affects up to 10% of chromatin [36].

In humans, a high percentage of sperm fragmentation, highlighted by TUNEL assay, appears to be associated with a significant reduction in pregnancy rate after IVF but not after ICSI [37]. On the contrary, a meta-analysis by Collins et al. shows a significant reduction in pregnancy rates in both IVF and ICSI cycles in cases of high DFI levels [38], whereas other meta-analyses report no interference of a high DFI with clinical outcome following ART [39].

Confirming the great variability in literature data between DFI and pregnancy rate, Avendano et al. have reported that in infertile men, spermatozoon with apparently normal morphology may have DNA fragmentation, and the presence of an increased proportion of normal spermatozoon with damaged DNA was negatively associated with embryo quality and pregnancy outcome after ICSI [40]. A similar result was obtained by Alvarez Sedo and coworkers, who demonstrated that in infertile patients with a DFI > 15%, there was a negative correlation between blastulation and pregnancy rates, compared with patients with DFI < 15%. They concluded that high levels of DNA damage promote embryo arrest and induce the activation of the apoptotic pathway [41].

In this retrospective study, we aimed to verify the relationship between the DFI and the human sperm morphology. This correlation was not observed in the whole semen sample, but only in the mobile sperm population, selected after pellet swim up technique that is normally used in embryology laboratories to recover mobile and morphologically good spermatozoa, among which select the single spermatozoon to be injected in ICSI treatment or to be used for fertilization in IVF treatment.

Our study was carried out on 125 patients allowed us to identify a 15% threshold above which DNA fragmentation is consistently present also in spermatozoa with normal morphology. The percentage of morphologically normal spermatozoa with altered chromatin in patients with DFI ≥ 15% was statistically higher than in patients with DFI < 15%.

To our knowledge, this is the first time that this information has been reported. The presence of a large number of spermatozoa with good morphology but fragmented DNA increases the risk that the oocyte, in particular with the ICSI technique that involves the intra-oocyte injection of a spermatozoon with good morphology, is fertilized by a spermatozoon carrying an altered chromatin, and despite the residual capacity of repair of the oocyte molecular machine, this condition can negatively affect the fertilization processes, the subsequent embryonic development, and the clinical outcomes in terms of a full-term pregnancy. In case of a high percentage of normal morphology spermatozoa with fragmented DNA, it would be common to envisage an increased risk of injecting a spermatozoon with abnormal chromatin, or stop the treatment and guide the patient towards a therapeutic treatment (e.g., antioxidants, gonadotropins), and repeat the TUNEL test to verify the reduction of sperm count with fragmented DNA.

For this reason, it seems rational to carry out the TUNEL assay on the selected mobile sperm population, where possible, collected after pellet swim up technique, in order to identify the probabilities of selecting a spermatozoon with altered chromatin. Obviously, the execution of the TUNEL assay on the whole semen sample remains a valid tool for diagnostic purposes in order to evaluate a correct spermatogenesis or the interference of chemical-physical factors that can alter the chromatin structure of the spermatozoa, not only to investigate male infertility in patients undergoing ART cycles but also in the workup of infertility and in abortions. In addition, our threshold of 15% in DFI, to evaluate a physiological condition in the sperm population, is identical or very close to that proposed by other authors, in particular Alvarez Sedò et al., that proposed a cutoff of 15% [41], and Hassanen et al., that proposed a cutoff of 20.3% [42]. The results of this study suggest to make more attention to normal spermatozoa of patients with a DFI ≥ 15% undergoing cycles of ICSI.

In conclusion, our data seem to demonstrate that in patients with DFI ≥ 15%, it is appropriate to perform also the DFI evaluation in spermatozoa isolated after a pellet swim up technique, to evaluate the risk of transferring, during the ICSI procedure, a spermatozoon whit normal morphology but fragmented DNA.

To this aim, it seems necessary to use a direct diagnostic technique with a high predictive capacity such as the TUNEL assay.

## Data Availability

Not applicable.
